# Safeguarding planetary and human health–reflections on the Virchow Prize 2024

**DOI:** 10.3389/fpubh.2025.1653872

**Published:** 2025-08-13

**Authors:** Ole Petter Ottersen, Victoria Grandsoult, Roland Göhde, Detlev Ganten

**Affiliations:** ^1^Department of Molecular Medicine, Institute of Basic Medical Sciences, University of Oslo, Oslo, Norway; ^2^Virchow Foundation, Berlin, Germany

**Keywords:** planetary health, health systems resilience, complex adaptive systems, Sustainable Development Goals (SDGs), health policy and systems research (HPSR), global health leadership, interdisciplinary public health

## Introduction

In this editorial, we briefly reflect on the Virchow Foundation and the Virchow Prize 2024 in light of the outstanding contributions of last year's laureates, Lucy Gilson and Johan Rockström.

More than ever, we now need a multipronged approach to health. Reducing health to a purely biomedical issue has never been more misjudged. Socioeconomic status, education, and community support are crucial determinants of health, as are environmental influences and climate change. A glance at the United Nations' 2030 Agenda of Sustainable Development Goals (SDGs) tells us the importance of seeing human health in the context of animal and planetary health and being duly attentive to the structural and systemic causes of poor health. A comprehensive and multifaceted approach to health requires social and political solutions, implying that health and wellbeing must be seen from a system perspective that transcends the reach of the healthcare sector. In simple words, to care for our health, we must go beyond healthcare.

The overarching motivation of the Virchow Prize is to promote “health for all” by honoring groundbreaking research, innovations, and other contributions toward the betterment of health on a global scale (1) (Supplementary Figure 1). “Health for all” implies reducing the health inequities that were deepened during the recent coronavirus disease 2019 (COVID-19) pandemic and that are now exacerbated by war and conflict, geopolitical tensions, erosion of multilateralism, and the rise of authoritarianism and protectionism. Importantly, “health for all” also emphasizes the need to care for the health of future generations. This is why the Virchow Prize is also set to recognize achievements in the realms of environmental and planetary health. We are on an unsustainable path that calls for collective action in the spirit of intergenerational justice. Crystallized in a few words: human health on a sick planet is a contradiction in terms.

Between them, the two Virchow Prize 2024 laureates represent the broad approach to health that the Virchow Prize strives to support. Lucy Gilson has championed health policy and systems research (HPSR), which seeks to “*understand and improve how societies organise themselves in achieving collective health goals, and how different actors interact in the policy and implementation processes to contribute to policy outcomes*.” Johan Rockström, on the other hand, has radically transformed our insight into the boundaries that define the planet's safe operating space. Together, they have set the scene for continued efforts to safeguard health for current and future generations.

The lectures delivered by Lucy Gilson and Johan Rockström in Berlin on 11 October 2024, on the occasion of the Virchow Prize ceremony, highlight the need for complementary approaches to health, climate, and the environment.

## Lucy Gilson and the relational fabric of health systems

Lucy Gilson's seminal contributions to HPSR offer a powerful complement to Rockström's macro-ecological focus. Grounded in the realities of health systems in low- and middle-income countries, Gilson conceptualizes these systems as complex, adaptive, and human-centered; shaped as much by relationships and power dynamics as by infrastructure or finance (2, 3).

Her scholarship emphasizes the relational elements of resilience, trust between health workers and communities, participatory governance, and inclusive leadership. These insights have deepened the understanding of why some health systems can adapt to shocks while others collapse, as evidenced during the COVID-19 pandemic and Ebola outbreaks (4).

Gilson's leadership in international consortia, such as Resilient and Responsive Health Systems (RESYST) and the Emerald Project, has shaped WHO guidance and strengthened the capacity for HPSR across sub-Saharan Africa and beyond. Her focus on “everyday resilience” underscores the importance of supporting frontline staff and community actors as core drivers of adaptive capacity (5).

## Johan Rockström and the science of planetary boundaries

Johan Rockström's research foregrounds the planetary boundaries' framework, which identifies nine critical Earth system processes essential for maintaining the planet's stability. These boundaries, ranging from climate regulation and biosphere integrity to biochemical cycles, define a “safe operating space for humanity” (6, 7).

Rockström's early study, with the Stockholm Resilience Centre and the Potsdam Institute for Climate Impact Research (PIK), demonstrated that multiple boundaries have already been breached, pushing Earth systems toward potentially irreversible tipping points. His more recent scholarship emphasizes how this environmental destabilization poses systemic risks to global health, including increased exposure to zoonotic diseases, food insecurity, and forced migration (7, 8).

Importantly, Rockström's contributions go beyond diagnostics. He promoted the “safe and just space for humanity” framework, integrating environmental ceilings with social foundations, thereby linking ecological sustainability to human equity (9, 10). This has informed policy across UN agencies, the Planetary Health Alliance, and the Lancet Commissions.

## Conclusion

Hosting the Virchow Prize in Berlin underscores Germany's ambition as a global health convener (11). It also aligns with the broader efforts to position Berlin as a hub for science-informed global health governance, exemplified by initiatives such as the World Health Summit, the Global Health Hub Germany, and the various activities in the area of international urban health.

As we face the cascading challenges of climate instability, health inequities, systemic fragility, and geopolitical tensions, the studies of Lucy Gilson and Johan Rockström illustrate the importance of freedom of research, scientific collaboration, and system-based public health in our efforts to improve health for all. Their scholarship illuminates paths toward resilience, both ecological and institutional, and calls on the global health community to bridge disciplines, embrace complexity, and act with foresight and compassion. Together, Gilson and Rockström's pioneering approaches catalyze progress toward the SDGs, while advancing equity for present and future generations and calling for a more resilient, healthier, and just world.

## References

[1] Oni T Ganten D Kampmann B Göhde R Ottersen OP. A prize for global health in the name of Rudolf Virchow. J Public Health Afr. (2023) 14:2862. 10.4081/jphia.2023.2862 37908388 PMC10615153

[2] Gilson L. Trust and the development of health care as a social institution. Soc Sci Med. (2003) 56:1453–68. 10.1016/S0277-9536(02)00142-9 12614697

[3] Sheikh K Gilson L Agyepong IA Hanson K Ssengooba F Bennett S. Building the field of health policy and systems research: framing the questions. PLoS Med. (2011) 8:e1001073. 10.1371/journal.pmed.1001073 21857809 PMC3156683

[4] Gilson L Barasa E Nzinga J Cleary S Goudge J Molyneux S. Everyday resilience in district health systems: emerging insights from Kenya. BMJ Glob Health. (2017) 2:e000224. 10.1136/bmjgh-2016-000224 29081995 PMC5656138

[5] Barasa E Mbau R Gilson L. What is resilience and how can it be nurtured? A systematic review of empirical literature on organizational resilience. Int J Health Policy Manag. (2018) 7:491–503. 10.15171/ijhpm.2018.06 29935126 PMC6015506

[6] Rockström J Steffen W Noone K Persson A Chapin FS Lambin EF. Planetary boundaries: Exploring the safe operating space for humanity. Nature. (2009) 461:472–5. 10.1038/461472a 19779433

[7] Richardson K Steffen W Lucht W Bendtsen J Cornell SE Donges JF. (2023). Earth beyond six of nine planetary boundaries. Sci Adv. (2023) 9:eadf0673. 10.1126/sciadv.adh2458 37703365 PMC10499318

[8] Steffen W Richardson K Rockström J Cornell SE Fetzer I Bennett EM. Planetary boundaries: guiding human development on a changing planet. Science. (2015) 347:1259855. 10.1126/science.1259855 25592418

[9] Rockström J Sukhdev P. How Food Connects All the SDGs. United Nations SDG Publication (2016). Available online at: https://www.stockholmresilience.org/research/research-videos/2018-08-22-how-food-connects-all-the-sdgs.html

[10] Raworth K. (2012). A Safe and Just Space for Humanity: Can We Live Within the Doughnut? Oxfam Discussion Paper (2012).

[11] Ganten D. Global Health – a challenge for sustainable development. J Mol Med. (2015) 93:235–41. 10.1007/s00109-015-1260-6

