# Supplementary material for: Safeguarding planetary and human health–reflections on the Virchow Prize 2024
Source: Front Public Health. 2025 Aug 13;13:1653872. doi: 10.3389/fpubh.2025.1653872 (PMC12380815; doi:10.3389/fpubh.2025.1653872)
Supplement: Supplementary file 1 [file Data_Sheet_1.docx]

Supplementary Material

Title: Safeguarding Planetary and Human Health – Reflections on the Virchow Prize 2024

 Authors: Ole Petter Ottersen, Victoria Grandsoult, Roland Göhde, Detlev Ganten


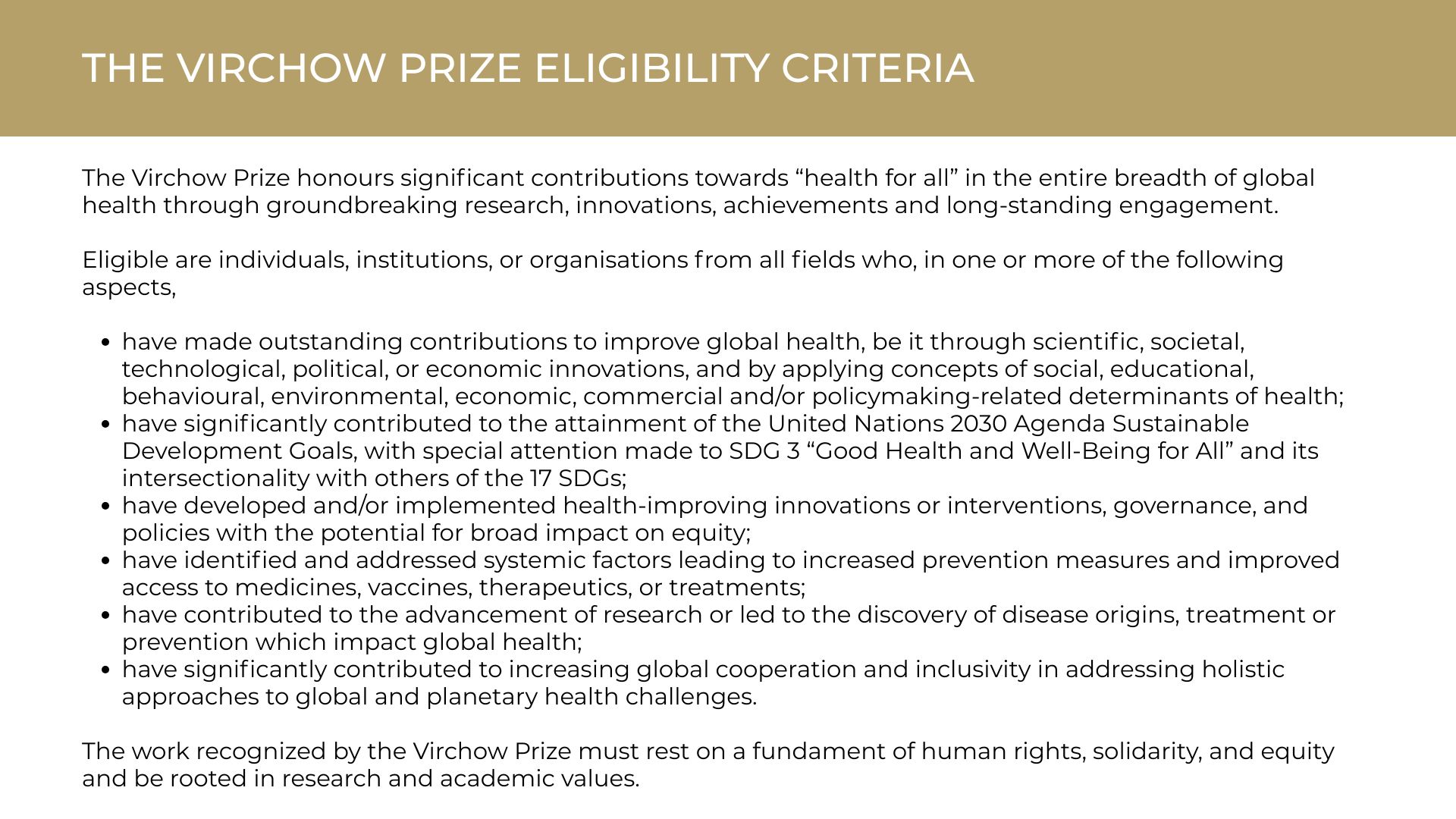


Supplementary Figure 1. *Eligibility Criteria for the Virchow Prize. https://virchowprize.org/nominations/*
